# Three *Leishmania* species identified in domestic dogs in Tunisia: New insights into canine leishmaniosis

**DOI:** 10.1371/journal.pntd.0014299

**Published:** 2026-05-11

**Authors:** Lilia Zribi, Maria Paola Maurelli, Mariele De Santi, Maria Ortensia Montella, Aida Bouratbine, Valentina Foglia Manzillo, Laura Rinaldi, Karim Aoun, Gaetano Oliva

**Affiliations:** 1 Lab of Medical Parasitology, Biotechnology & Biomolecules, Institut Pasteur de Tunis, University of Tunis El-Manar, Tunis, Tunisia; 2 Department of Veterinary Medicine and Animal Production, University of Naples “Federico II, Naples, Italy; Tokat Gaziosmanpaşa University Faculty of Medicine: Tokat Gaziosmanpasa Universitesi Saglik Bilimleri Fakultesi, TÜRKIYE

## Abstract

**Background:**

Tunisia represents the perfect example of a Mediterranean Country where different *Leishmania* species may express their infectivity causing Visceral leishmaniasis by *Leishmania* (*L*.) *infantum*, Zoonotic Cutaneous Leishmaniasis by *L*. *major* and chronic cutaneous leishmaniasis by *L. tropica*. The recent detection of *L. major* in two dogs living in Tunisia confirms how this animal may host both visceral and cutaneous *Leishmania* species. The present study reports the results of 4 field surveys performed in central and southern districts of Tunisia: Zaghouan (ZA); Kairouan (2 surveys, K1 and K2); Tataouine (TA), to assess the prevalence of *Leishmania* species in dog.

**Methods:**

One hundred and sixteen dogs were enrolled. Blood, lymph node and skin samples were collected with theowner’ consent. Thirty-two were enrolled during 2021 in ZA (n = 32), fifty-four were enrolled in KA during 2022 (K1; n = 22) and 2024 (K2, n = 32) and thirty were enrolled in TA during 2024 (n = 30). All dogs correspond to new surveys other than those investigated in a previous study. In total 218 biological samples were analyzed by qPCR (kDNA), end-point PCR (ITS-1) and nested-PCR (SSUrRNA). The purified positive PCR products were sequenced. All dogs were classified as asymptomatic or with mild clinical signs, not specifically attributable to Canine Leishmaniosis due to the presence of fleas and tick infestation.

**Results:**

Thirty-eight dogs tested positive by molecular techniques (32.75%%). *Leishmania infantum* was the most identified species (31/116, 26.72%). Extremely high prevalence was found in K2 (23/32, 71.87%) compared with the previous study K1 (10/22, 45.45%). One dog (K1) was positive to *L. tropica,* the first detection of this species in Tunisia, while two dogs (ZA and K2) confirmed the presence of *L. major*. Interestingly, we found for the first time a dog positive to *L. infantum/donovani* in TA, an arid area where no VL cases have been previously recorded.

**Conclusions:**

This study pointed out the high circulation of *L. infantum* in north and central Tunisia and underlines as the dog can host all the 3 *Leishmania* species present in this country.

## Author summary

The epidemiological data on canine leishmaniasis in Tunisia is outdated and does not reflect the changes in distribution that have been described worldwide. The present study demonstrates that dogs are capable of hosting all three *Leishmania* species *(Leishmania infantum, Leishmania major, Leishmania tropica)* occurring in this country, the agents of visceral and cutaneous forms of Leishmaniasis in humans. Our study is interesting because it is the first time that a study has been conducted in southeastern Tunisia, particularly in Tataouine, where the disease had never been described before. We have succeeded in demonstrating that *L. infantum* parasite circulates in the region and can potentially cause the disease, both in humans and in dog and cat. This may be an important indication for veterinarians and health authorities in the region, who should no longer rule it out in cases of suspicion. In addition, our study focused on another region, Zaghouan, located in the north of the country, where canine leishmaniasis caused by *L.infantum* has a very high prevalence. Moreover, this is the first time that a team has described *L.tropica* in a dog in Tunisia. This study also confirms, as already described in a previous study by our team, that *L. major* can be found in the skin of dogs.

## Introduction

**Fig 1:**
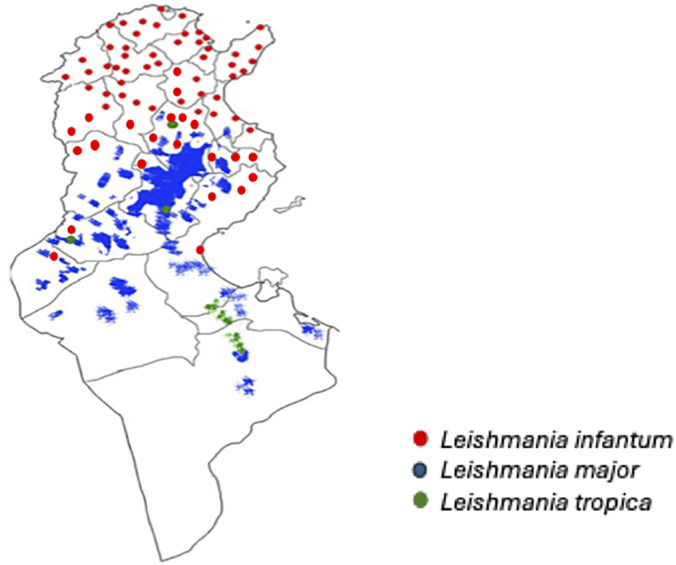
Distribution of Leishmania species in Tunisia.

Tunisia represents the perfect example of a Mediterranean Country where different *Leishmania* species coexist and express their infectivity in humans causing Visceral Leishmaniasis (VL) by *Leishmania* (*L*.) *infantum*, Zoonotic Cutaneous Leishmaniasis (ZCL) by *L*. *major* and chronic cutaneous leishmaniasis (CCL) by *L. tropica* [1–3]. *Leishmania infantum* is also considered the agent of sporadic cutaneous clinical cases (Sporadic Cutaneous Leishmaniasis, SCL) [4]. Dogs are considered the main domestic reservoirs of *L. infantum* that causes Canine Leishmaniosis (CanL) in this animal [5].The recent detection of *L. major* in two dogs living in Tunisia confirms that this animal can host both visceral and cutaneous *Leishmania* species [6]. The geographical distribution of the three *Leishmania* species has been documented since many years, with *L. infantum* limited to the Northern and Central regions of the country and *L. major* and *L. tropica* endemic in the Center and South of the country [1,3,7,8] (Fig 1) Southern Tunisia is characterized by extreme aridity, with a desert climate and low annual rainfall (mean = 110 mm). In this region, clinical cases of *L. infantum*, both visceral and cutaneous, have not been reported, except for few reports in the oasis of Tozeur (South-West Tunisia), where more favorable microclimatic conditions exist [2,9]. The geographical distribution of CanL overlaps that of VL, with no reported cases from most southern areas of the country [2,5]. For these reasons infected dogs can be used as indicators to i) confirm the presence of *L. infantum* in previously known endemic areas; ii) detect significant variation in CanL prevalence to alert Health Authorities; iii) detect the presence of *L. infantum* in regions where there are not reported human cases; iiii) assess the presence of *Leishmania* species other than *L. infantum* in dog*.* This present study is an update of the epidemiological data of CanL in Tunisia based on the results of recent field surveys performed in 3 different governorates of Northern on (Zaghouan – ZA), central (Kairouan – KA), and southern (Tataouine – TA) regions of the country.

## Methods

### Ethics statement

All procedures involving animals were conducted in accordance with the ethical guidelines and principles for international animal welfare. The study protocol was approved by the Animal Ethics Committee - National School of Veterinary Medicine, Sidi Thabet Approval Number: 79-CEEA-ENMV. All procedures were designed to minimize pain and suffering. Informed consent was obtained from the owner.

**Fig 2:**
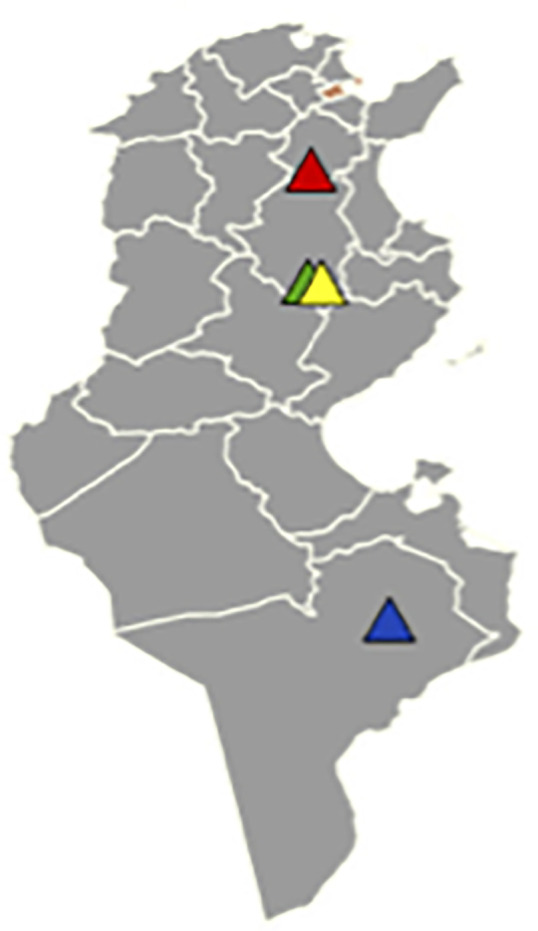
Tunisia map with sampling geographical areas. **(Orange**: Tunis, capital of Tunisia, **Red**: Zaghouan area, **Green** and **Yellow**: Kairouan1 and 2 areas, **Blue**: Tataouine area) https://gadm.org/download_country.html.

A total of 116 dogs were enrolled in this study. Thirty-two were enrolled during 2021 in ZA (n = 32), fifty-four were enrolled in KA during 2022 (K1; n = 22) and 2024 (K2, n = 32) and thirty were enrolled in TA during 2024 (n = 30) (Fig 2). All dogs correspond to new surveys other than those investigated in a previous study [10]. Most dogs lived outdoor and their age ranged from one to ten years. After clinical examination, dogs were classified as asymptomatic or symptomatic in presence of one or more clinical signs compatible with CanL. Three ml of blood was collected from cephalic vein and split in two different tubes, one containing EDTA and another with serum separator gel. When present, skin lesions (SL) were sampled by using cytology brushes, lymph nodes (LN) samples were obtained by fine needle aspiration from one or both popliteal LN, when easily sampleable. Samples were collected following the Good Clinical Practice medical procedures and animal welfare. Tubes were kept at +4 °C during field collection (2–3 days). EDTA and whole blood tubes were centrifuged for 10 min at 2500 rpm to separate red blood cells, buffy coat (BC) plasma and serum. Serum and plasma were stored at−20 °C until serological analysis, while BC, LN and skin samples were stored at−20 °C until DNA extraction. In total, 218 biological samples were collected. In detail, DNA was extracted from 112 BC, 70 LN, and 36 SL samples using the DNeasy Blood and Tissue kit (Qiagen, Leipzig, Germany) according to the manufacturer’s instructions. Subsequently, three PCR methods were employed for analysis of all DNA samples: (i) qPCR to amplify a region of the minicircle kinetoplast DNA (kDNA) [11]; (ii) end-point PCR to amplify the Internal Transcribed Spacer 1 (ITS-1) [12]; (iii) nested PCR (n-PCR) to amplify the small subunit ribosomal RNA (SSUrRNA) [13]. In each PCR run, positive and negative controls provided by the National Reference Center for Leishmaniosis (CReNaL, Palermo, Italy) were used. Details of the three amplification protocols (i.e., primers, concentrations of PCR reagents conditions, thermal cycling conditions) are described in Maurelli et al. [6].

PCR products obtained from ITS-1 and SSUrRNA amplifications were visualized on a 2% low melting agarose gel stained with ethidium bromide (Bio-Rad, USA) and purified using the QIAquick Gel Extraction KIT (Qiagen, Germany). Purified amplicons were sequenced in both forward and reverse directions and analyzed using the Chromas software (version 2.6.6). Sequence identities were determined using BLASTn (https://blast.ncbi.nlm.nih.gov/Blast.cgi). Only samples that yielded a clear sequence were considered positive.

Serological diagnosis was performed by enzyme-linked immunosorbent assay (ELISA) ID Screen Leishmaniasis Indirect Test kit (ID vet, Innovative diagnostics, France), previously used in similar studies [6,10]. Sera of infected dogs were also tested with a rapid test, SNAP 4Dx Plus Test (IDEXX Laboratories Inc., Westbrook, Maine, USA) to detect possible co-infections with other Canine Vector Borne pathogens (CVBp), specifically *Anaplasma phagocytophilum/platys, Ehrlichia canis/ewingii, Dirofilaria immitis, Borrellia burgdorferi.*

## Results

*Leishmania* spp. DNA was found in 38 dogs, six of which were also positive by ELISA. Three dogs resulted positive by ELISA but negative by PCR methods. Overall, 41 of 116 dogs (35.34%) were retained as infected. Extremely high prevalence was found in K2 (23/32, 71.9%) compared with K1 (10/22, 45.45%, p < 0.001). Statistical significance was also found between ZA (18.8%) and K2 and K1 and TA (6.7%) (p < 0.001 and p = 0.042, respectively) Based on clinical examination positive dogs were classified as asymptomatic or with mild clinical signs (47.5%), primarily weight loss, lymph node enlargement and cutaneous lesions. These signs could not be specifically attributed to CanL for the presence of fleas and tick infestation and in absence of exclusion of other diseases, mainly intestinal worms. After sequencing of PCR products, most dogs (30, 25.86%) were infected by *L. infantum* (GenBank Access numbers: ITS-1 KY963126; SSUrRNA MK495995), while two (1.72%) by *L. major* (GenBank Access number: ITS-1 MN60413; SSUrRNA MZ520152) and one (0.86%) by *L. tropica* (GenBank Access number: PX230125). DNA of the remaining three *Leishmania* PCR positive dogs could not be definitively identified: three were classified as *Leishmania infantum/donovani* (GenBank Access number: KR081262/ KT438661) and two as *Leishmania* spp*.* (GenBank Access number: PX088761). Among the 218 biological samples, 24.10% of BC resulted positive, while LN and SL were 12.85% and 33.33%, respectively. ITS1 was the most sensitive PCR method (p = 0.0007 and p = 0.004, McNemar test) when compared to n-PCR and qPCR respectively. Complete results are reported in the Table 1. DNA of the two cutaneous isolates corresponded to *L. major* (ZA and K2) while *L. tropica* DNA was found in BC of a K1 dog. Skin lesions of the two *L. major* positive dogs were limited to small alopecia areas of the ear pinna, not typically resembling the skin lesions described in human cases of ZCL [3]. Thirty out of forty infected dogs (80%) tested positive for one or more CVBp. To rule out the possible interference of co-infections with the state of *Leishmania* spp. infection, the sera from 37 negative dogs were randomly selected and tested with the same rapid test. Twenty-eight (75.7%) resulted positive for at least one CVBp, with no statistical difference with the *Leishmania* infected dogs (p = 0.961, X^2^ test). The investigated sites confirmed the presence in dog of the 3 *Leishmania* species recognized in Tunisia for human beings. Table 2 summarized the results of infected dogs in the four study sites.

**Table 1:** Infected dogs: positive results in the different analyzed specimens.

Studyarea	Dog ID	Buffy coat	Lymph node	Skin	Species	Elisa
ZA	26	POS^a^			*L. infantum*	
	27	POS^a^			*L. infantum*	
	34	POS^a^			*L. infantum*	
	57					POS
	61					POS
	66			POS^b^	*L. major*	
K1	1	POS^b^	POS^b^ POS^c.^		*L. infantum*	POS
	3		POS^b^ POS^c^		*L. infantum*	POS
	5	POS^b^ POS^a.^			*L. infantum*	
	6	POS^b^ POS^a.^			*L. infantum*	
	7	POS^b^ POS^a^ POS^c^			*L. infantum*	
	8	POS^a^ POS^c.^			*L. infantum*	
	17		POS^b^ POS^c^		*L. infantum*	POS
	19		POS^b^ POS^c^		*L. infantum*	POS
	20	POS^b^			*L. infantum*	
	21	POS^b^			*L. tropica*	
K2	3	POS^c^		POS^b^	*L. infantum/L.donovani*	POS
	4	POS^b^		POS^b^	*L. infantum*	
	5	POS^b^ POS^c^			*L. infantum*	
	6	POS^b^			*L. infantum*	
	7	POS^b^		POS^c^	*L. infantum*	
	*9*		POS^a^ POS^c.^		*L. infantum*	
	12	POS^b^		POS^b^	*L. infantum*	
	13			POS^b^ POS^a^ POS^c^	*L. infantum*	
	14			POS^b^ POS^a.^	*L. infantum*	
	15			POS^b^ POS^a.^	*L. infantum*	
	16	POS^b^		POS^b^ POS^c^	*L. infantum*	
	17	POS^b^			*L. infantum/L. donovani*	
	20			POS^b^	*L. infantum*	
	22	POS^b^	POS^c.^		*L. infantum*	
	23	POS^b^			*L. infantum*	
	24	POS^b^			*L. infantum*	
	25	POS^b^	POS^c.^	POS^a^ POS^c.^	*L. infantum*	
	27	POS^b^ POS^c^			*L. infantum*	
	28	POS^b^			*L. infantum*	
	29	POS^b^			*L. infantum*	
	30	POS^b^			*L. infantum*	
	31		POS^a^ POS^c.^		*Leishmania spp.*	
	32			POS^a^ POS^c.^	*L. major*	
TA	17					POS
	22	POS^b^			*L. infantum/ L. donovani*	POS

Legend: POS^b^ = ITS1; POS^a^ = nPCR; POS^c^ = qPCR; ZA = Zaghouan; K1 and K2 = Kairouan 2022 and 2024; TA = Tataouine.

**Table 2:** Infected dogs and *Leishmania* species found in the four field studies.

Study area (year)	n.dogs	n. infected (%)	Leishmania species (n.)
ZA (2021)	32	6* (18.75)	*L. infantum* (2) *L.major* (1) *Leishmania* spp. (1)
K1 (2022)	22	10 (45.45.90)	*L. infantum* (9) *L. tropica* (1)
K2 (2024)	32	23 (71.87)	*L. infantum* (19) *L. infantum/donovani* (2) *L. major* (1) *Leishmania spp.* (1)
TA (2024)	30	2** (6.66)	*L. infantum/donovani* (1)
**TOTAL**	**116**	**41 (35.3)**	***L. infantum* (30) *L major* (2) *L. tropica* (1)** ***L. infantum/donovani* (3) *Leishmania spp.* (2)**

*Two dogs resulted positive to ELISA but negative to the 3 PCR methods.

**One dog resulted positive to ELISA but egative to the 3 PCR methods.

## Discussion

Three species of the *Leishmani*a genus are endemic in Tunisia [1,3,8] causing infection in humans and animals. The geographical distribution of *L. infantum* is limited to the northern and central regions of the country with very few reports in the microclimate area of the oasis of Tozeur in the Southwest [8,9]. Many authors have considered that the extreme aridity of southern Tunisia could represent an ecological barrier preventing the transmission of *L. infantum* [14]. *Leishmania major and L. tropica* are endemic in the Center and South of the country [1,3,7,8]. In the governorate of Tataouine, Southeastern Tunisia, only cases of Cutaneous Leishmaniasis (CL) due to *L. major* and *L. tropica* are reported [1,2,15]. While the dog is the reservoir of *L. infantum,* wild rodents act as reservoirs of *L. major* and *L. tropica*, respectively *Psammomys obesus* and *Meriones shawi*, and *Ctenodactylus gondii* [16–18].Infectiousness of *L. infantum* infected dogs to competent phlebotomine vectors has been associated with many factors, the main being the severity of the disease. The evaluation of dog’s infectiousness remains still difficult due to the need of xenodiagnosis as the only valid method to assess it [19]. Although, serological and specific PCR detect *L. infantum* infection in dogs appear weakly useful to provide information on infectiousness, particularly in asymptomatic animals. However, they represent indubitable methods to assess the presence of the parasite that makes dogs as important epidemiological sentinel. The increasing dog population and the growing relationship between dogs and humans, are considered two focal points in the complex epidemiology of leishmaniosis. Preventative and therapeutic strategies are the only useful methods to limit the spread of parasite from infected dogs to other animal reservoirs and to susceptible human population. Overlapping outbreaks of human and canine VL have been reported in the North regions of Tunisia, where *L. infantum* is the dominant species both in visceral and cutaneous cases [2,5]. A recent study underlined a high seroprevalence of CanL (58.3%) in 317 dogs from the northern Tunisia [20]. *Leishmania* DNA was found in 21.2% of dogs, using blood samples tested by end-point PCR targeting the 145 bp conserved region of *L. infantum* kinetoplast DNA [21]. This high seroprevalence seems indicate the increasing of CanL in the most populated area of Tunisia, compared with previous data showing around 20% prevalence in the same regions [22]. Many factors may explain the increasing of CanL and VL, the main are the agricultural expansion and breeding practices that contribute to the development of favorable environments for phlebotomine colonization [14]. In addition, the lack of control plan for CanL, particularly the not systemic application of sand fly bites preventive tools, such as pyrethroid topic compounds or impregnated collars, both in owned and stray dogs, together with the limited treatment of sick dogs, have contributed to CanL spread. The overlap between canine and human leishmaniasis was also demonstrated in our previous study carried out in two neighboring governorates of central Tunisia, Zaghouan and Kairouan, where VL and ZCL human cases are predominant, respectively [3,23]. Interestingly, the study evidenced higher prevalence (27%) of CanL in the areas where VL is more frequent than ZCL [10]. In the present study, while CanL prevalence in Zaghouan was confirmed around 20%, there was a surprising increase of CanL in Kairouan governorate, where it reached over 70% during 2024. This increase is not easy to explain, mainly in a region traditionally considered endemic for ZCL, with an annual incidence ranging from 200 to 600 cases [3]. High CanL prevalence in Kairouan could be the consequence of the looseness of the sand fly control campaigns, continued urbanization of the rural and suburban areas, land use changes and climatic change that allow the parasites and their vectors spreading in space and time as it was noticed in Europe [24]. Twenty-one sand fly species have been recorded in Tunisia, belonging to *Phlebotomus* (n.13) and *Sergentomia* (n.8) genera [25], with the presence of *P. perniciosus, P. papatasi and P. sergenti*, competent vectors of *L. infantum, L. major* and *L. tropica* widely demonstrated in our study areas [14,26–29]. Most positive cases were diagnosed from BC samples, where blood is usually considered less sensitive than LN and bone marrow [30]. Differences in sensitivities reported by many authors for the *Leishmania* DNA detection may depend by several factors, such as the PCR technique, differences in the study populations, the clinical condition of dogs and the CanL prevalence rates that may conditionate parasite burden [31]. In the present study, three different PCR methods have been performed, to increase the probability of finding positive samples and to characterize the *Leishmania* species. Quantitative real-time is not optimal to characterize the *Leishmania* species. For this reason, we also used two end-point PCRs targeting two different targets (SSUrRNA and ITS-1), because in literature conflicting opinions are reported regarding the sensitivity of these two targets. Indeed, some authors report higher sensitivity for SSUrRNA target [12,32], whereas others favor the ITS-1 [33,34]. The detection of parasite in bone marrow and lymph nodes result typically positive in dogs with CanL and can also be used for detection of *Leishmania* in asymptomatic infected seronegative dogs [35,36], this finding may explain why many PCR positive dogs resulted negative to ELISA test. The evidence of 3 serologically positive dogs with no direct demonstration of Leishmania by PCR is a condition that is already demonstrated. In a longitudinal study performed in Italy for three consecutive sand fly seasons, some asymptomatic infected dogs diagnosed by bone marrow (BM) n-PCR converted to a negative status and were classified as “transient” n-PCR positives. The possible explanation was that after infection, the parasite load in BM dropped to undetectable for a variable period of time until the number of organisms increased again [37].

A further longitudinal study performed in Italy [38] suggested that BC nested PCR positivity may be used as early marker of infection, with their possible negativization during the time. In the same study, conjunctival swabs were classified as a persistent marker, also in absence of seroconversion. In our study, the most positive BC samples have been collected during February (K2) and March (K1), two months that are considered free of sand fly activity, for this reason positive blood PCR may indicate the evidence of parasitemia 3–4 months after the end of sand fly activity season and infective bites. Other vector-borne diseases may also contribute to the development and clinical evolution of CanL [39,40]. In the present study, the 80% of *Leishmania* spp. positive dogs were found co-infected with one or more others transmitted pathogens, mainly *Ehrlichia canis/ewingii* and *Anaplasma phagocytophilum/platys.* However, the influence of co-infections cannot be demonstrated due to the presence of tick and fleas infestation in all selected dogs, causing CVBPs serological positive results also in *Leishmania* negative dogs.

The demonstration of two *L. major* positive dogs confirms that dogs can harbor this *Leishmania* species, as previously described [6]. While one positive dog was from the same recorded area, Kairouan governorate, we also found the first *L. major* positive dog in Zaghouan governorate, where VL is more frequently reported than ZCL. This finding adds new evidence on the northward spread of ZCL in Tunisia [2,3].

This study also describes the first case of *L. tropica* in a dog living in Tunisia. This species was already reported in dogs in other countries, like Israel [41], Yemen [42], Iran [43, 44], Pakistan [45] Turkey [46]; Saudi Arabia [47], both in symptomatic and asymptomatic dogs. As for *L. major* canine cases, the epidemiological role of dogs as primary reservoirs of this cutaneous *Leishmania* species needs to be further investigated. However, it is enough evident that the dog may host multiple *Leishmania* species in the same environmental context, that makes it a good indicator for the circulation of this complex parasite in a determined region. From an epidemiological point of view, it is also important to point out the first record of two *Leishmania* positive dogs in Tataouine, south Tunisia, where this species had never been reported before in human or animals. This evidence may indicate the continuous southward spread of *L. infantum* in Tunisia and the need of more accurate diagnostic etiological approach in patients with cutaneous or systemic symptoms related to *Leishmania* infection.

## Conclusion

In conclusion, our study highlights the high circulation of *L. infantum* in all Tunisia territory and emphasizes that the dog can host all the three *Leishmania* species present in this country.

## Supporting information

S1 File Data. (XLSX)
